# Unique Location and Origin of a Ganglion Cyst

**DOI:** 10.7759/cureus.16453

**Published:** 2021-07-18

**Authors:** Mohammad U Khubaib, Robert Monaco

**Affiliations:** 1 Sports Medicine, Active Orthopedics & Sports Medicine, Hackensack, USA; 2 Sports Medicine, Atlantic Sports Medicine, Morristown, USA

**Keywords:** ganglion cyst, biceps ganglion cyst, bicipital groove, long head of biceps, sports medicine, non-surgical orthopedics, sonography, ultrasound-guided, aspiration, pain management

## Abstract

Ganglion cysts usually occur around the wrist. Occasionally, they can also occur around the shoulder in the spinoglenoid and suprascapular notches. Rarely they can be found on the long head of biceps as it traverses the glenohumeral joint. Such lesions are usually diagnosed on MRI and might need minor surgery. We present the case of a young athlete with a rare location of ganglion cyst, successfully diagnosed and managed non-invasively by using sonography. This represents a time and cost-conscious approach as compared to traditional methods.

## Introduction

Ganglion cysts are articular or tendon-sheath lesions possibly originating as a result of herniation of the synovium or arising from the tendon fibrils. They occur mostly in women aged 30-50 years, most commonly around the wrist and are occasionally associated with trauma as well [1]. Some ganglion cysts can be found in the vicinity of the shoulder. When this happens they are usually found in the spinoglenoid and suprascapular notches [2]. Rarely they can be found along the long head of the biceps tendon, usually as it traverses the glenohumeral joint. There have been a few case reports/series about biceps ganglion cyst in the shoulder joint visualized on MRI, but no reported cases exist of the use of ultrasound for these. We report a unique case of a 13-year-old female gymnast who was found to have a ganglion cyst in the bicipital groove managed through sonography.

## Case presentation

A 13-year-old female gymnast with no significant medical history was referred to sports medicine by her primary care physician for right shoulder pain ongoing for six months. The symptoms might have been triggered after a week of aggressive CrossFit training. The pain had two components to it: posterior shoulder pain due to a snapping scapula (which is beyond the scope of this article), and more significantly, dull and achy pain in the front of her shoulder, radiating down her biceps, worsened with lifting weights and shoulder extension, and relieved by rest. The patient had tried nonsteroidal anti-inflammatory drugs (NSAIDs), rest, and activity modification with minimal relief. 

Physical examination revealed a well-developed and well-nourished female in no apparent distress. The head, eyes, ear, nose, and throat (HEENT), respiratory, cardiovascular, abdominal, skin, vascular, and neurological examinations were within normal limits. Inspection of the right shoulder revealed no bruising, rash or visualized swelling. Palpation elicited no pain over the deltoid, acromioclavicular joint, clavicle, sternoclavicular joint, coracoid or the pectoralis minor. There was tenderness to palpation over the right proximal biceps with the patient complaining of some radiation down the biceps as well. There was periscapular pain on palpation of the right scapula as well. Both shoulders had normal strength and range of motion in all planes but moderate scapular dyskinesis was noted. The patient demonstrated positive Hawkin’s, Mayo Shear, Speed’s and Empty Can tests on the right side. Orthopedic examination of the other joints was within normal limits. 

**Figure 1:**
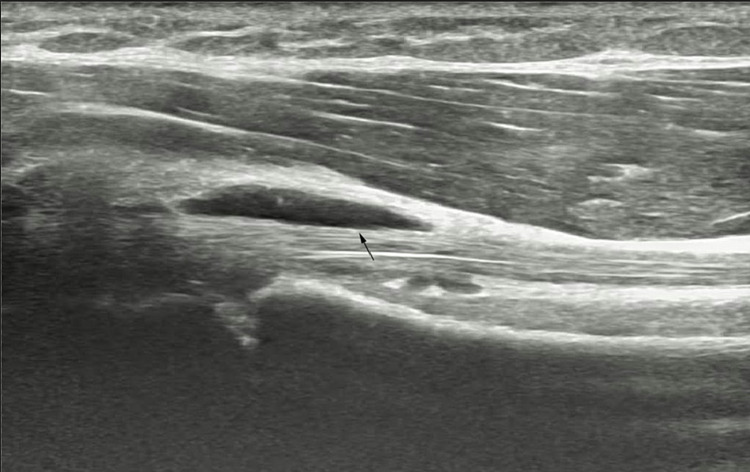
Black arrow pointing to the ganglion cyst next to the biceps tendon.

**Figure 2:**
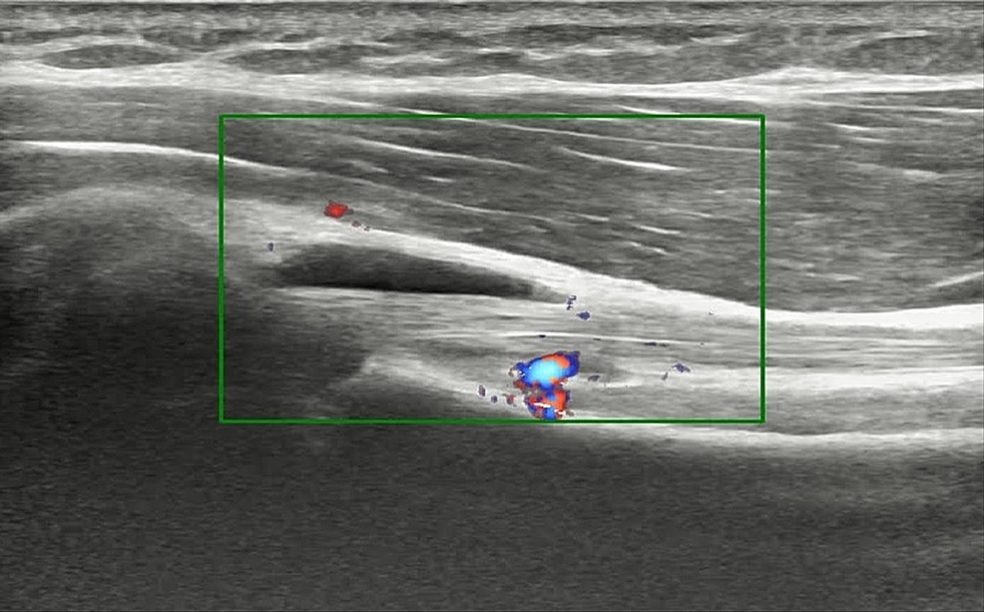
Doppler sonogram showing a negative signal within the lesion.

A magnetic resonance imaging (MRI) scan revealed the long head of the biceps tendon to be intact, normal in signal, and normal in position. However. a 12x6x23 mm lobulated collection was visualized along the long head of the biceps tendon in the bicipital groove. Differential diagnosis included a ganglion cyst versus focal tenosynovitis. A linear probe ultrasound was used to visualize the lesion which appeared to be hypoechoic and was found to be minimally compressible (Figure 1) and having a negative signal on Doppler imaging (Figure 2). 

After discussions with the patient regarding possible management options, the lesion was approached under ultrasound guidance along the long axis in the lateral to medial direction as the patient lay supine with the arm at 90 degrees to the body. The humeral circumflex artery was visualized inferolaterally and was avoided. Lidocaine was administered with a 25 gauge needle in the surrounding tissues and 0.5 cc of thick mucous fluid was aspirated from the ganglion. The cyst was punctured and injected with 2.5 cc of lidocaine and 40 mg of methylprednisolone. Post-procedure, immediate pain relief was experienced. 

On a follow-up appointment three months later, the patient had significant pain relief during activities and no pain was elicited on physical examination. Repeat sonographic imaging showed a small incompressible remnant of the lesion. Repeat MRI six months after the procedure was within normal limits.

## Discussion

On ultrasound, a ganglion cyst appears as a hypoechoic/anechoic lesion with well-defined margins and occasional internal septations. On MRIs, it appears as a unilocular or multilocular rounded or lobular fluid signal mass, adjacent to a joint or tendon sheath [3]. Both of these findings, as well as the thick mucoid consistency of the fluid aspirated from our patient’s lesion, favor a diagnosis of ganglion cyst rather than focal tenosynovitis [4]. The proximal upper extremity is a rare location for a ganglion cyst and one associated with the long head of the biceps is even more infrequent. A ganglion cyst inside the bicipital groove has only been reported once [2], but what makes this case truly unique is the successful management via a relatively conservative approach of ultrasound-guided aspiration rather than invasive management. The case also demonstrates the clinical yield of sonography in not only diagnostic but therapeutic management as well. While MRIs are frequently used to diagnose shoulder joint pathology, the application of an ultrasound probe can save time and money, while simultaneously offering a management option and immediate relief in selected cases.

## Conclusions

Ganglion cysts are soft tissue lesions usually found around the wrist. It is extremely rare for a ganglion cyst to be associated with the biceps tendon. When this occurs, it can cause symptoms of impingement and injury to the rotator cuff or biceps. Ultrasound can be of high diagnostic and therapeutic yield in such cases, while also conserving time and money.
